# Exome sequencing-based identification of novel type 2 diabetes risk allele loci in the Qatari population

**DOI:** 10.1371/journal.pone.0199837

**Published:** 2018-09-13

**Authors:** Sarah L. O’Beirne, Jacqueline Salit, Juan L. Rodriguez-Flores, Michelle R. Staudt, Charbel Abi Khalil, Khalid A. Fakhro, Amal Robay, Monica D. Ramstetter, Joel A. Malek, Mahmoud Zirie, Amin Jayyousi, Ramin Badii, Ajayeb Al-Nabet Al-Marri, Abdulbari Bener, Mai Mahmoud, Maria J. Chiuchiolo, Alya Al-Shakaki, Omar Chidiac, Dora Stadler, Jason G. Mezey, Ronald G. Crystal

**Affiliations:** 1 Department of Genetic Medicine, Weill Cornell Medical College, New York, New York, United States of America; 2 Division of Pulmonary and Critical Care Medicine, Department of Medicine, Weill Cornell Medical College, New York, New York, United States of America; 3 Department of Genetic Medicine, Weill Cornell Medical College-Qatar, Doha, Qatar; 4 Division of Translational Medicine, Sidra Medical Research Centre, Doha, Qatar; 5 Department of Biological Statistics and Computational Biology, Cornell University, Ithaca, NY, United States of America; 6 Department of Medicine, Hamad Medical Corporation, Doha, Qatar; 7 Laboratory Medicine and Pathology, Hamad Medical Corporation, Doha, Qatar; 8 Department of Medicine, Weill Cornell Medical College-Qatar, Doha, Qatar; Wake Forest School of Medicine, UNITED STATES

## Abstract

**Background:**

Type 2 diabetes (T2D) susceptibility is influenced by genetic and lifestyle factors. To date, the majority of genetic studies of T2D have been in populations of European and Asian descent. The focus of this study is on genetic variations underlying T2D in Qataris, a population with one of the highest incidences of T2D worldwide.

**Results:**

Illumina HiSeq exome sequencing was performed on 864 Qatari subjects (574 T2D cases, 290 controls). Sequence kernel association test (SKAT) gene-based analysis identified an association for low frequency potentially deleterious variants in 6 genes. However, these findings were not replicated by SKAT analysis in an independent cohort of 12,699 exomes, primarly due to the absence of low frequency potentially deleterious variants in 5 of the 6 genes. Interestingly one of the genes identified, catenin beta 1 (*CTNNB1*, β-catenin), is the key effector of the Wnt pathway and interacts with the nuclear receptor transcription factor 7-like 2 (*TCF7L2*), variants which are the most strongly associated with risk of developing T2D worldwide. Single variant analysis did not identify any associated variants, suggesting the SKAT association signal was not driven by individual variants. None of the 6 associated genes were among 634 previously described T2D genes.

**Conclusions:**

The observation that genes not previously linked to T2D in prior studies of European and Asian populations are associated with T2D in Qatar provides new insights into the complexity of T2D pathogenesis and emphasizes the importance of understudied populations when assessing genetic variation in the pathogenesis of common disorders.

## Introduction

The prevalence of type 2 diabetes (T2D) in Qatar is one of the highest in the world [1]. T2D is a complex disease with both inheritance and superimposed environmental factors such as diet and lifestyle playing a role in susceptibility to the condition [2,3]. Genetic studies in T2D including genome-wide association studies (GWAS) and exome sequencing have helped to explain the inherited basis and pathogenesis of the condition and identified genes that influence pancreatic beta-cell function/insulin secretion and insulin resistance [4–6]. However, the prevalence of T2D varies widely among populations and despite the growing epidemic of T2D in the Middle East, the majority of these studies have been carried out in populations of European or Asian descent, populations where the prevalence of T2D is much lower than in Qatar [1,6]. Given genetic diversity among populations, the information from GWAS findings in Europeans or Asians may not be transferable to other populations including the Qataris.

We have previously demonstrated that the common T2D risk alleles identified in the European and Asian populations do not replicate in the Qatari population [7]. In the present study, exome sequencing of 864 Qataris (approximately 0.25% of the entire Qatari population) was applied for comparison of 574 Qataris with T2D to 290 Qatari non-diabetic controls. Using sequence kernel association analysis, low frequency (0.01 to 0.1 minor allele frequency (MAF)) potentially deleterious variants associated with T2D were identified in 6 genes, including catenin beta 1 (*CTNNB1*, β-catenin) the key effector of the Wnt pathway, which interacts with the nuclear receptor transcription factor 7-like 2 (*TCF7L2*), the gene most strongly associated with risk of developing T2D worldwide [8]. Additionally, *CTNNB1* interacts with the Wnt pathway member dishevelled segment polarity protein 1 (*DVL1*), also identified in this analysis. However, it was not possible to replicate the study findings in an independent cohort of 12,699 exomes made up of individuals from various populations in the T2D-GENES cohort [9], downloaded with permission from the European Genome Archive (https://www.ebi.ac.uk/ega/studies/EGAS00001001460) and analysed using the identical pipeline.

## Subjects and methods

### Study population

Under protocols approved by the Institutional Review Boards of Hamad Medical Corporation (HMC) and Weill Cornell Medical College Qatar (WCMC-Q), subjects were recruited from HMC clinics and written informed consent obtained. A total of 864 subjects (574 cases with T2D and 290 controls) were included in the study (S1 Table). Most of the subjects were participants in our previous study [7]. T2D was diagnosed based on the American Diabetes Association (ADA) criteria including fasting blood glucose ≥126 mg/dL and/or 2 hour plasma glucose ≥200 mg/dL during an oral glucose tolerance test and/or HbA1C ≥6.5% [10]. All subjects were over the age of 30 with a family history of a minimum three generations of ancestry in Qatar. Detailed subject assessment is described in this article’s S1 File.

### Sequencing and variant detection

Exome sequencing was performed at the New York Genome Center (NYGC) as previously described [11]. Reads were mapped to the GRCh37 human reference genome and prepared for variant calling using GATK best practices [12], and a call set for all 864 exomes was produced by simultaneous genotyping using the GATK UnifiedGenotyper algorithm [12]. Data quality was optimized and verified based on multiple metrics described in S1 File. Variants (n = 295,515) were categorized based on MAF into singleton (MAF <0.001, n = 46,874), rare (MAF 0.001 to 0.01, n = 190,771, including n = 64,258 doubletons), low-frequency (MAF 0.01 to 0.1, n = 58,671) and common (MAF >0.1, n = 46,073). Singleton and rare variants were excluded from the SKAT analysis due to the enrichment of false positive variants in this dataset, as demonstrated in our prior study of the same exome dataset *versus* whole genome sequence data [11]. Additionally, common variants were excluded as our prior study demonstrated that they are not associated with T2D in Qatar [7]; leaving n = 58,671 variants.

Variants in protein coding genes were identified using SnpEff v.4.2 [13] which uses ENSEMBL v.75 gene models to assign variants to genes, and to determine variant functional region and impact on the assigned gene. SnpEff classified variants in protein coding genes into 4 impact categories (modifier, low, moderate and high) based on their potential for altering protein structure and function. The focus of this study was on variants of moderate and high potential for deleteriousness, which included missense and loss of function (LoF) single nucleotide variants (SNV). In addition, quantitative scores of deleteriousness were calculated using CADD v.1.3 [14]. Variants were also annotated with respect to allele frequency in the cohort, cases, controls, 1000 Genomes Phase 3 v.5 [15] and ExAC v.3.1 [16], calculated using VCFTools v.0.1.14 [17].

### Statistical analysis

To identify genes linked to diabetes most effectively, the analysis was limited to low frequency (MAF 0.01 to 0.1) variants with moderate or high potential for altering protein structure or function (n = 20,492). Four distinct association analyses were conducted on the whole-exome sequence data genotypes, including gene-based analysis (sequence kernel association test, SKAT) [18] and variant-based single variant analysis (SVA) [19], with Bonferroni multiple testing correction for all genes and for known T2D genes [20]. The genetic models tested included an association test for each variant after applying prior filters (SVA), and an association test for each gene (SKAT) containing at least 1 variant after filtering. The SVA was conducted using EMMAX [19], with age, gender, body mass index (BMI) and kinship matrix (calculated by EMMAX-KIN) as covariates. The SKAT test was conducted using the SKAT v.1.2.2 (https://cran.r-project.org/web/packages/SKAT/) library in R v.3.3.2. (https://cran.r-project.org/). Two gene set filters were considered- all protein coding genes, and a subset of tested genes overlapping with 634 genes previously linked to T2D (Supplemental Table 20 of Fuchsberg *et al* [20]). These gene sets were used for multiple testing corrections by the Bonferroni method [21] with alpha = 0.05. Population structure was accounted for in the SKAT and SVA analysis using the kinship matrix, with kinship analysis conducted using EMMAX-KIN v.10Mar2010 [19]. While no relatives were knowingly included in the analysis, the Qatari population has a high prevalence of consanguineous marriage [19,22], increasing the likelihood that relatives may have been included inadverently.

The Qatari population can be divided into 3 major ancestry clusters (Arab/Bedouin (Q1), Persian/South Asian (Q2) and Sub-Saharan African (Q3)) [11,22,23]. A majority of individuals in this study belonged to the Q1 (n = 605) and Q2 (n = 210) clusters, with a smaller number (n = 49) from the Q3 subpopulation, proportions similar to those observed in the general Qatari population. Consanguinity is high and comparable to other Middle Eastern populations in the Q1 and Q2 subpopulations, while consanguinity is lower and more comparable to African populations in the Q3 subpopulation [24]. The relatively higher consanguinity among the Q1 and Q2 individuals included in this study was confirmed in another study which reconstructed large pedigrees of 1^st^, 2^nd^, and 3^rd^ cousins among n = 1376 Qataris including the n = 864 Qataris from this study [11]. A kinship matrix was utilized to control for population structure, accounting for both near and distant relationships (including consanguineous subpopulations). Adequate population structure correction was verified by QQ plots of the variant distributions. Although no “genomic control” lambda adjustment was conducted, the lambda value was calculated for both the SKAT and SVA distributions [25], to assess inflation due to residual population structure or polygenic disease risk [26].

To exclude the possibility of gaps in coverage depth for significant genes, the coverage depth mean and 95% confidence interval was calculated for each exon, in each individual, in each significant gene using Samtools with exons defined by the Agilent target list bed file, and summary of the results was calculated in R.

### Replication

To replicate any associations identified in this study in an independent cohort, SKAT test p values were calculated for 12,699 exomes from the T2D-GENES cohort [9,20], obtained with permission from the European Genome Archive (https://www.ebi.ac.uk/ega/studies/EGAS00001001460). SKAT was calculated for each gene that was associated with T2D in the analysis using the identical code and parameters used for analysis of Qatari subjects.

### Investigation of known T2D loci

Prior studies have identified at least n = 81 variants linked to T2D in one or more human populations [20]. To determine whether any of these variants are linked to T2D in Qatar, confidently genotyped variants that overlapped with the set of n = 81 were extracted from the n = 295,515 genotyped in Qataris by exome sequencing. The case and control allele frequency for known alleles was compared between published reports related to the identified variants in the Qatari data. In addition, the power to detect an association in Qatar in the current study was calculated for each of the identified variants (S6 Table) using Purcell’s Genetic Power Calculator [27], assuming the same odds ratio as in published studies [28–32], as previously described [7].

### Data sharing

All sequence read data and individual and population VCF files were submitted to the Sequence Read Archive (SRA) section of the NCBI SRA database (SRA accession #SRP061463, BioProject 288292). In addition, phenotype and covariate data and the population VCF was made available through our website http://geneticmedicine.weill.cornell.edu/genome.html. Code for replicating the analysis was also made available (https://github.com/juansearch/_WAS).

## Results

### Demographics

A total of 864 subjects passed quality control, all were over the age of 30 and self-reported to have three generations of native Qatari ancestry. Of these, 574 were classified as T2D cases based on ADA criteria for fasting plasma glucose, oral glucose tolerance test and/or HbA1C level, as detailed in S1 File. The controls (n = 290) had normal fasting glucose and HbA1C ≤6.5%. The majority of the subjects were female both for cases with T2D (344/574, 60%) and controls (169/290, 58%; p>0.5). T2D cases were significantly older with a mean age of 56 ± 10 yr *vs* 46 ± 9 yr in controls (p<10^−10^). BMI was significantly higher in T2D cases (33 ± 7 kg/m^2^) compared to controls (31 ± 7 kg/m^2^; p<10^−4^). HbA1C was significantly higher in T2D cases (8.4 ± 1.9%) compared to controls (5.7 ± 0.4%, p<10^−10^; S1 Table).

### Association tests

Both gene-based sequence kernel association test (SKAT) and variant-based SVA association tests were conducted on potentially deleterious low frequency variants. After variant filtering based on allele frequency (1% to 10%) and function (missense or LoF), exome sequencing identified a total of n = 20,492 potentially deleterious SNVs in n = 9,378 protein coding genes (S4 Table). These variants were tested for association with T2D using the SKAT with age, gender, BMI and population structure as covariates. Rather than exclude relatives, the kinship matrix of all 864 Qataris was used to control for population structure (S1 Fig). Population structure was also examined using Principal Components Analysis of Qataris (S2A Fig) and Qataris with 1000 Genomes Populations (S2B Fig), and confirmed to show the range of diversity previously described in this population [11,23]. Two multiple testing corrections were applied, limiting the analysis to either all genes or known T2D genes [20], combined with application of Bonferroni [21] multiple testing correction.

Six genes had Bonferroni-significant p values in the SKAT analysis (S2 Table). None of the associated genes were among 332 known T2D genes tested, and Bonferroni multiple testing correction limited to known T2D genes did not identify any significant genes. In addition, SVA did not identify any individual variants associated with T2D by either multiple testing correction. The lack of significant SVA results could be attributed to low power given the sample size, as well as prior studies showing the lack of replication for T2D SVA hits in Qatar [7].

**Fig 1:**
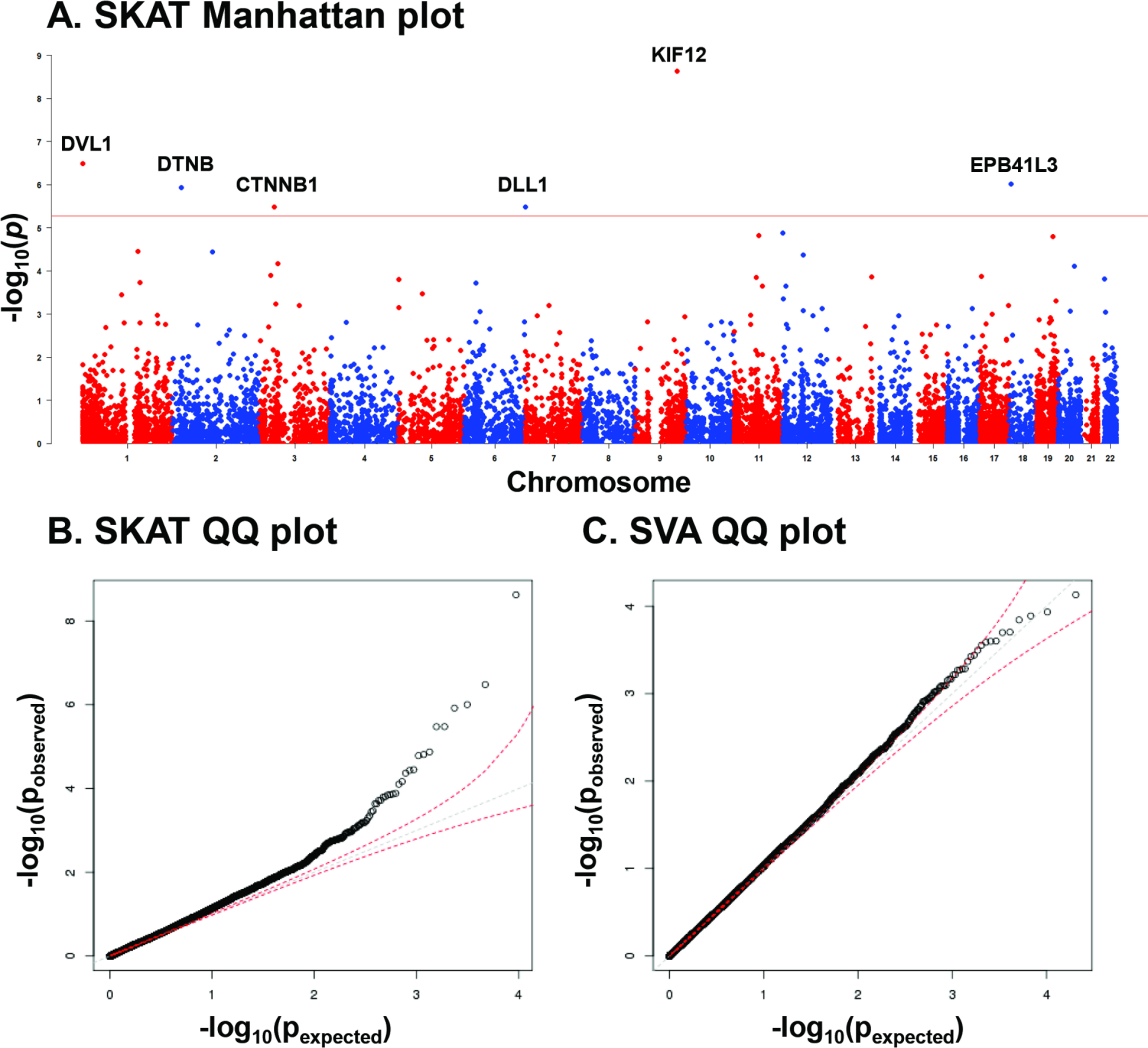
Manhattan and quantile-quantile (QQ) plots of genome-wide sequence kernel association analysis (SKAT) and QQ plot of single variant analysis (SVA) of 20,492 low-frequency potentially deleterious variants in 9,378 genes in all 864 Qataris (574 cases with type 2 diabetes (T2D) and 290 controls), with age, gender, BMI and kinship as covariates. Six genes were significant in the analysis, with p values below Bonferroni multiple testing threshold (alpha = 0.05). **A.** Manhattan plot of genome wide association analysis. The plots show–log_10_ (p value) on the *y-*axis and the chromosomal position of each variant on the *x-*axis. Genes are ranked by uncorrected p values. Genes with p < the Bonferroni correction p value threshold are labeled in the plot [above the red horizontal line, 1og_10_(0.05/22003) = 5.64]. **B.** QQ plot between observed and expected p values for SKAT. The red broken lines represent the confidence band indicating the range of results consistent with a 95% interval around the null (grey broken line). No lambda correction was applied. The observed lambda value was lambda = 1.15 for SKAT. **C.** QQ plot between observed and expected p values for SVA of potentially deleterious variants using EMMAX [19], with age, gender, BMI and kinship as covariates. The red broken lines represent the confidence band indicating the range of results consistent with a 95% interval around the null (grey broken line). No lambda correction was applied. The observed lambda value was lambda = 1.03 for SVA.

The 6 genes that were significant under Bonferroni multiple testing correction in the SKAT analysis of potentially deleterious low frequency variants included *CTNNB1*, *DLL1*, *DTNB*, *DVL1*, *EPB41L3*, and *KIF12* (Table 1, Fig 1A.). The QQ plot for the analysis showed no evidence of major inflation in SKAT analysis (lambda = 1.15), with no need for lambda adjustment (Fig 1B), and similar findings for SVA of the same variants (lambda = 1.03) (Fig 1C). Of 9 potentially deleterious low frequency variants in these 6 genes, 8 had combined annotation dependent depletion (CADD) scores >9 (Table 2). Coverage depth mean and 95% confidence interval across exons in these 6 genes was 60.8 ± 0.27, excluding gaps in coverage depth that could produce false positive or false negative variants. None of the genes replicated in the T2D-GENES cohort (S3 Table), primarily due to lack of potentially deleterious low frequency variants in this cohort for 5 of 6 genes. The only gene where a replication test was possible was *EPB41L3*, which contained 4 potentially deleterious low frequency variants (p > 0.81).

**Table 1:** Qatari type 2 diabetes (T2D) at-risk genes identified by sequence kernel association test (SKAT) of low-frequency potentially deleterious protein coding single nucleotide polymorphisms (SNP)^1^.

Gene	Gene name	Chr	Start	End	Potentially deleterious coding variants^2^	SKAT p^3^	Significance threshold
*KIF12*	Kinesin family member 12	9	114,091,623	114,100,099	1	2.37x10^-9^	Bonferroni
*DVL1*	Dishevelled segment polarity protein 1	1	1,335,278	1,349,142	1	3.30x10^-7^	Bonferroni
*EPB41L3*	Erythrocyte membrane protein band 4.1 like 3	18	5,392,381	5,630,666	3	9.91x10^-7^	Bonferroni
*DTNB*	Dystrobrevin beta	2	25,377,220	25,673,647	2	1.20x10^-6^	Bonferroni
*DLL1*	Delta like canonical Notch ligand 1	6	170,282,200	170,291,075	1	3.34 x10^-6^	Bonferroni
*CTNNB1*	Catenin beta 1	3	41,199,451	41,240,448	1	3.35 x10^-6^	Bonferroni

^1^ The SKAT analysis presented was conducted on all 864 Qataris, limited to 20,492 potentially deleterious (missense or loss of function, LoF) low-frequency (minor allele frequency 1% to 10%) variants in 9,378 protein coding genes. None of these genes were previously linked to T2D, and the known T2D genes [20] with the lowest p value was CCAAT/enhancer binding protein alpha (*CEBPA*), with a p value of 2.93x10^-4^. A total of 54 genes had p-values lower than *CEBPA*, including the 6 shown.

^2^ Potentially deleterious defined as missense or LoF.

^3^ SKAT p: Sequence kernel association test p value for the gene.

**Table 2:** Single nucleotide polymorphisms (SNP) in qatari type 2 diabetes (T2D) at-risk genes^1^^,^^2^.

Gene										Genotype frequencies						Minor allele frequency^3^			
										Cases			Controls						
	Chr	Pos	rsID	SVA p value	CADD^4^	Transcript change	Protein change	Function	Minor/ Major alleles	Hom^5^ Minor (cases)	Het^6^ (cases)	Hom Major (cases)	Hom Minor (controls)	Het (controls)	Hom Major (controls)	Cases	Controls	ExAC	1000 Genomes
*KIF12*	9	116,859,679	.	0.8899	28	c.134T>C	p.Leu45Pro	Missense	G/A	0	3	571	0	25	265	0.0026	0.0431	0.0000	0.0000
*DVL1*	1	1,271,676	.	0.4723	9	c.1934A>C	p.His645Pro	Missense	G/T	0	16	558	0	36	254	0.0139	0.0621	0.0000	0.0000
*EPB41L3*	18	5,397,367	.	0.0145	10	c.2531T>C	p.Leu844Pro	Missense	G/A	0	14	560	0	34	256	0.0122	0.0586	0.0000	0.0000
*EPB41L3*	18	5,416,160	rs8082898	0.6001	9	c.1724A>G	p.Tyr575Cys	Missense	C/T	0	16	558	0	7	283	0.0139	0.0121	0.0340	0.0565
*EPB41L3*	18	5,478,295	rs117900256	0.6378	23	c.326G>T	p.Ser109Ile	Missense	A/C	1	37	536	0	16	274	0.0340	0.0276	0.0130	0.0122
*DTNB*	2	25,611,134	.	0.5382	25	c.1672A>C	p.Thr558Pro	Missense	G/T	0	11	563	0	31	259	0.0096	0.0535	0.0000	0.0000
*DTNB*	2	25,611,140	rs562264712	0.5472	23	c.1666A>C	p.Thr556Pro	Missense	G/T	0	37	537	0	40	250	0.0322	0.0690	0.0000	0.0116
*DLL1*	6	170,592,620	rs200861263	0.0460	0	c.1747T>C	p.Cys583Arg	Missense	G/A	0	61	513	0	69	221	0.0531	0.1190	0.0002	0.0000
*CTNNB1*	3	41,278,119	rs77750814	0.5528	23	c.1995C>A	p.Asp665Glu	Missense	A/C	0	75	499	0	6	284	0.0653	0.0103	0.1090	0.0000

^**1**^ Single variant analysis (SVA) was conducted to identify associations between low frequency potentially deleterious variants and T2D using EMMAX v.10Mar2010 on all 864 Qataris, using age, gender, BMI and a kinship matrix calculated using EMMAX-KIN as covariates. To determine if single variants in the 6 associated genes (Table 1) were driving the SKAT association signal, the SVA p values are presented for potentially deleterious variants in these genes. Variants were functionally annotated using SnpEff v.4.2 using ENSEMBL v.75 gene models, and potentially deleterious variants were either missense or loss of function variants.

^**2**^ Shown (from left-to-right) is the gene symbol, chromosome (Chr) and position (Pos) of the variant, DbSNP v.147 rsID for the variant (or “.” if novel), the SVA p value, combined annotation dependent depletion (CADD) score, transcript change (in reference-alternate allele order), protein change (in reference-alternate allele order), variant function, minor and major alleles, genotype counts for cases and controls, minor allele frequency in cases, controls, ExAC and 1000G.

^**3**^ The Qatari minor allele frequency was quantified in ExAC v.0.3.1 [16] and in 1000 Genomes Phase 3 v.5 [15].

^**4**^ CADD scores were calculated for each variant to further assess the potential for deleteriousness [14].

^5^ Hom: Homozygous

^6^ Het: Heterozygous

### Integration of SKAT and SVA results

Though SVA did not identify variants significantly associated with T2D in Qatar, it provided information on the directionality of variant effect for the 9 variants identified within the 6 SKAT-significant T2D associated genes, based on variant frequency in cases and controls. In the case of 6 of the 9 variants, the minor allele in Qatar had a higher frequency in controls and appeared to be protective (*KIF12* p.Leu45Pro, *DVL1* p.His645Pro, *EPB41L3* p.Leu844Pro, *DTNB* p.Thr558Pro, *DTNB* p.Thr556Pro, and *DLL1* p.Cys583Arg), while the other 3 variants (*EPB41L3* p.Tyr575Cys, *EPB41L3* p.Ser109Ile and *CTNNB1* p.Asp665Glu.) appeared to be risk increasing with a higher frequency in cases.

### Investigation of known T2D loci

While no single variant was significant after multiple testing correction, the allele frequency in cases and controls was compared to prior studies for n = 6 variants previously linked to T2D [20,29–32] located within protein coding exons genotyped in this study. These variants included well-known T2D associated variants, such as the *PPARG* p.Pro12Ala variant (rs1801282) that was previously shown not replicate in Qataris [33] (S5 Table). For four of the six variants (67%) the known risk allele from prior reports was the major allele and, in five of the six variants (83%), the risk allele had a higher allele frequency in cases not surprising given the high prevalence of T2D in Qatar. The lowest p value among the 6 variants was for rs515071 (p < 0.036), which is located near an intron/exon junction of ankyrin 1 *(ANK1)*. While the expected odds ratio in the Qatari population is unknown, assuming the same odds ratio as published studies and given the sample size, this study had insufficient power to detect an association for these 6 variants in single variant analysis (S6 Table), confirmed by the lack of significant SVA associations. However, the advantage of SKAT in this study is that it combines data across variants in the same gene, which leads to additional power to detect an association and a less stringent multiple testing correction.

## Discussion

The prevalence of T2D in Qatar is one of the highest in the world [1]. In the present study, exome sequencing, a strategy not previously applied in the study of T2D in Middle Eastern populations, identified 6 genes (*KIF12*, *DVL1*, *EPB41L3*, *DTNB*, *DLL1*, *CTNNB1*) with potentially deleterious low frequency variants significantly associated with T2D. Interestingly, none of these genes replicated in an independent cohort of 12,699 exomes from T2D-GENES. This observation provides new insights into the complexity of the pathogenesis of T2D and emphasizes the importance of understudied populations. Out of 6 genes associated with T2D, 5 contained potentially deleterious variants present only in Qataris, highlighting the unique genetics of T2D in this population.

### Prior studies of T2D risk alleles in middle eastern populations

To date, GWAS performed mostly in European and Asian populations, have identified over 80 loci associated with T2D that include 634 genes [4–6,34,35] and exome sequencing has identified over 10 T2D risk genes [4–6,20,36]. However, these associations may not replicate in other populations due to variability in risk allele frequency in the control and/or the specific phenotype being assessed [37]. There have been no prior GWAS or exome studies of T2D risk alleles in Middle Eastern populations. We previously reported that from a panel of 37 single nucleotide polymorphisms (SNP) commonly associated in GWAS of T2D in Europeans and Asians, only two SNPs in *TCF7L2*, rs7903146 and rs4506565 were associated with T2D in Qataris, suggesting that the genetic risks for T2D are different in Qataris compared to Europeans and Asians [7]. Further, candidate gene studies of T2D in Middle Eastern populations yielded inconsistent results. While it was reported that many of the known European risk alleles were associated with T2D in Lebanese Arabs [38], the Saudi Arabian population [39], and in Tunisians and Moroccans [40], others did not demonstrate an association between the European T2D risk SNPs in these populations. For example, though a link between SNPs in *TCF7L2* and T2D was reported in Moroccans [40], only a marginal association was found in Arabs [41] and several European SNPs were not associated with T2D in Tunisians [42]. Finally, only 2 of 23 loci associated with BMI in other populations were linked to obesity in Qataris [43].

### Exome analysis in the Qatari population

Exome sequencing identifies low frequency population specific coding variants that would not otherwise be detected by array genotyping, with the potential to explain some of the heritability not identified by GWAS [44,45]. Out of n = 81 SNPs associated with T2D by GWAS [20], very few have led to the discovery of causal variants [44,46]. In contrast, exome sequencing assesses only the coding portion of the genome, and when the analysis is limited to potentially deleterious (missense and LoF) variants that alter protein function, the associated variants have a plausible mechanism of functional importance [47]. However, unless sample or effect sizes are very large, most statistical tests are underpowered to identify rare variants. To circumvent this challenge, SKAT an association test, which uses a multiple regression model to test for association between multiple variants in a sequenced region and a phenotype, was developed [18]. Recent updates to SKAT allowed adjustment for covariates and kinship in the statistical model. Furthermore, in contrast to burden tests, SKAT allows different variants in this study to have positive, negative or no effect on the phenotype [18]. Using SKAT, this study identified 6 genes with potentially deleterious low frequency variants associated with T2D in the Qatari population. These genes have not previously been linked to T2D risk in GWAS or exome sequencing analysis of European or Asian populations, confirming prior studies failing to replicate known T2D risk alleles in the Qatari population [7]. For 5 of the genes potentially deleterious variants were identified in Qataris only, with no potentially deleterious variants observed in the large T2D-GENES cohort used for replication testing [9].

The gene most strongly associated with T2D in Qataris and replicated in other populations was *DVL1*. This gene encodes a member of a family of intracellular scaffolding proteins that act downstream of transmembrane Wnt receptors playing an important role in the signalling pathway including interacting with and stabilizing *CTNNB1*. It was previously linked to autosomal dominant Robinow syndrome 2, [48] and exhibited an accelerated rate of evolution in primates as compared to rodents, most prominent in the lineage from primates to humans [49]. With regards to diabetes, methylation at CpGs in the body of *DVL1* has been associated with the development of T2D [50], and expression of *DVL1* is reduced in adipose tissue of non-diabetic subjects with insulin resistance compared to controls [51]. In addition, *DVL1* was previously identified within a quantitative trait locus region for T2D [52].

*CTNNB1* (previously known as β-catenin) mediates canonical Wnt signalling, a pathway critically involved in many processes including embryogenesis, cell growth and motility [53]. Wnt signalling is tightly regulated during growth and development of the pancreas and islet cells, and the Wnt pathway is involved in pancreatic beta-cell proliferation, glucose homeostasis and lipid metabolism [53]. Interestingly *TCF7L2* is a nuclear receptor for *CTNNB1*, and *TCF7L2* variants are the most strongly associated with risk of developing T2D, and have been reproduced in many populations [8,53], including Qataris [7]. Additionally, prior studies have demonstrated that during WNT signalling, *DVL1* a component of the pathway acts to stabilize *CTNNB1* leading to accumulation of *CTNNB1* in the nucleus and subsequent transcription of Wnt-responsive genes [54]. Activation of the Wnt/β-catenin pathway has been demonstrated to improve pancreatic beta-cell regeneration in diabetic rats [55], a discovery with potential therapeutic implications in T2D.

As with the Wnt pathway, Notch signalling has been shown to be critical for normal pancreatic development, controlling differentiation of pancreatic progenitor cells into endocrine and exocrine cells [56], and has also been implicated in the development of T2D. *DLL1* is a delta-like Notch ligand. Notch signalling has been demonstrated to control development of the murine endocrine pancreas, with *DLL1* knockout mice displaying premature endocrine cell development and subsequent pancreatic hypoplasia [57]. *In vitro* induction of Delta-Notch pathway ligand expression including *DLL1* in adult human pancreatic cells leads to the development of insulin expressing cells [58]. Additionally, a SNP in an intra-genic region spanning several genes including *DLL1* has been associated with type 1 diabetes (T1D) [59]. Also in T1D, it was demonstrated that *DLL1* protein expression is elevated in smooth skeletal muscle in cases compared to controls [60].

Kinesin family proteins (KIF) are a group of proteins with motor and microtubule cytoskeleton functions. Members of this superfamily are recognized to play an important role in the control of blood sugar both through pancreatic beta-cell insulin secretion and insulin stimulated glucose uptake in target tissues [61]. The *KIF12* gene codes for a cytoplasmic scaffold protein involved in microtubule motor functions. *KIF12* is expressed in fetal liver, adult brain and pancreatic islets, as well as renal tumors, and pancreatic cancer. Lipotoxicity due to chronic exposure to excess fatty acids, as may occur in obesity, is believed to damage pancreatic beta-cell *via* increased oxidative stress leading to beta-cell dysfunction and diabetes [62]. In the pancreas and kidney, *KIF12* expression is induced by the hepatocyte nuclear factor (*HNF)1-α/1β* [63], which in turn is inactivated by fatty acids [64]. *HNF1α*, is an activator encoded by the most frequently mutated gene in human monogenic diabetes (MODY3) [65]. *KIF12* has been demonstrated to mediate an antioxidant cascade in beta-cells in mice, and *KIF12* knockout mice have impaired glucose-induced insulin secretion and increased beta-cell oxidative stress [66]. This gene potentially links dietary fatty acids and obesity to increased oxidative stress in beta-cell leading to beta-cell dysfunction and T2D.

*EPB41L3* (DAL-1, protein 4.1B) is a member of the membrane/cytoskeleton-associated protein 4.1 superfamily, and has not previously been linked to T2D. In addition to acting as a membrane cytoskeleton component, it is believed to function as a tumor suppressor, and is downregulated in several cancers including non-small cell lung cancer and breast cancer [67]. It has been linked to pancreatic beta-cell carcinoma in mice [68], however its role in the condition in humans remains unknown. *EPB41L3* inhibits cell proliferation and promotes apoptosis by modulating the activity of proteins including protein arginine methyltransferase 3 (*PRMT3*) [67,69]. Interestingly, *PRMT3* has been demonstrated to play a role in mycophenolic acid induced pancreatic beta-cell apoptosis *in vitro* [70].

*DTNB* is a component of the dystrophin-associated protein complex, which acts as a scaffold for signalling proteins, with abnormalities in this complex leading to muscular dystrophy [71]. It is expressed predominantly in the brain and has not previously been linked with T2D.

### Study limitations

The main limitations of this study can be grouped into three categories: population sampling, genetic assay selection, and analytical model selection. With respect to population sampling, the cohort sampled in this study is small relative to recently published exome cohorts where T2D-associated genes were not identified. Given the small size of the cohort, it is challenging to argue that this study could find an association where larger studies did not. However, a recent population-specific study finding potentially deleterious variants linked to T2D in Greenland [36] suggests that there remain novel and population-specific undiscovered T2D-associated loci. By focusing on low frequency potentially deleterious variants in the exome of Qataris, this study provides additional evidence that population-specific variants are linked to T2D.

With respect to genetic assay selection, exome sequencing was utilized to identify low frequency and population specific variants, based on the knowledge from targeted genotyping that known T2D loci do not replicate in the Qatari population [7], and that Qataris variants are poorly represented on conventional genotyping arrays [11]. While rare variants are also of interest, a larger cohort would have been required to sample these variants with high confidence, as singletons in exomes were enriched with false positives [11]. The focus in this study was on potentially deleterious variants in protein coding exons, as these variants have a clear impact on protein function. While off-target and non-coding (intronic, regulatory) variants represent the majority of common variants linked to T2D [20], and can also be sampled by exome sequencing, variant detection false positive rates increase outside target regions and require stringent filters that can produce false negatives in regions of interest [72]. Additionally, this study excluded common variants, contrary to the common-disease-common-variant hypothesis that the same variants and the same genes are associated with T2D in every human population [73]. Given the evolutionary history of human populations, including the isolated evolution of the Arab/Bedouin (Q1) and Persian/South Asian (Q2) Qatari sub-populations (which represent the majority of Qataris in this study and in the general population) for tens of thousands of years [24], the accumulation of recent variants in the Qatari population that influence metabolism is plausible [74].

The associated genes in this study did not replicate in the T2D-GENES cohort [9], primarily due to the lack of potentially deleterious variants in 5 of the 6 associated genes. However, from a broader perspective there are 3 different ways to consider replication- variant replication, gene replication and pathway replication. The original form of replication is variant replication, where a variant associated with T2D in for example a European population replicates in the Qatar population, a scenario that this prior study failed to observe [7]. The second form of replication is gene replication, where different variants in the same gene lead to the same phenotype- loss of function of a protein. This also was not observed in the current study, due to the great genetic distance between the populations compared, and accumulation of low-frequency variants over time. However, our study did observe pathway replication, whereby various genes in the same pathway were linked to T2D in different studies. One of the genes identified, catenin beta 1 (*CTNNB1*, β-catenin), interacts with the nuclear receptor transcription factor 7-like 2 (*TCF7L2*), variants in which are the most strongly associated with risk of developing T2D worldwide. Thus it appears that Qataris have variants which alter the function pathways associated with T2D, however these variants are located different genes from other populations.

Finally, with respect to analytical model limitations, the current study chose the SKAT method, correcting for population structure using a kinship matrix, and including the covariates of age, gender and BMI. Based on the QQ plots observed, population stratification correction was adequate, and appears to account for both near and distant relationships. The diversity of the Qatari population is a known issue, as the Arab/Bedouin (Q1) and Persian/South Asian (Q2) clusters are characterized by high inbreeding, while the Sub-Saharan African (Q3) cluster exhibits low consanguinity and reflects more recent migration to Qatar [24]. As a further argument supporting the of use of kinship matrices for population stratification, our prior study comparing the Qatari population in comparison to 1000 Genomes [24] reconstructed the demographic history of human migration out-of-Africa using a neighbour-joining tree of the kinship matrix, comparable to the degree of resolution obtained using principal components analysis [75]. In addition, the kinship matrix of exome data for 1376 Qataris enabled reconstruction of extended families among Arab/Bedouin (Q1), Persian/South Asian (Q2), and Sub Saharan African Qataris (Q3), identifying 1^st^, 2^nd^, and 3^rd^ degree relationships not known prior to the study [11]. The SKAT model used here was limited with respect to computation, which scaled on the order of the cube of the sample size. Thus, analysis of only the 6 genes significantly associated in Qatar was conducted on the T2D-GENES cohort. Faster versions of SKAT are in development and could be applied in future studies.

### Conclusions

The genetic architecture of T2D is heterogeneous, and can be explained by variants across the spectrum of allele frequency and function. Based on prior studies by this group and others, and confirmed in this study, known T2D loci did not replicate in Qatar. The most likely scenario to explain this lack of replication is the unique genetics of the Qatari population, the majority of whom (Q1 and Q2 subpopulations) have lived in genetic isolation for thousands of years practicing within-tribe consanguineous marriage. As a result, risk alleles for T2D were not found to be shared between Qataris and other populations. This study confirms the hypothesis of population-specific T2D risk loci by applying exome sequencing and SKAT analysis, identifying 6 novel genes linked to T2D only in Qatar. Replication in the T2D-GENES cohort failed, primarily due to the lack of potentially deleterious variants in the same genes in this cohort. Future studies in larger cohorts of closely related Arab populations will hopefully confirm the unique architecture of T2D in a region with high disease prevalence.

## Supporting information

S1 Table Demographics of Type 2 diabetes cases and controls. (PDF)

S2 Table Results of Type 2 diabetes association analyses conducted on 864 qataris. (PDF)

S3 Table Replication of sequence kernel association test (SKAT) significant genes by burden test in an independent cohort. (PDF)

S4 Table Potentially deleterious low frequency snps in qataris. (PDF)

S5 Table Known protein coding loci linked to Type 2 diabetes. (PDF)

S6 Table Power calculation for known protein coding loci linked to type 2 diabetes. (PDF)

S1 Fig Kinship distribution. In order to account for population structure and relatedness in the analysis, kinship was calculated for each pair of Qataris (n = 864 individuals) using KING v.2 [27]. Shown is the frequency distribution of kinship scores, where higher numbers on the *x*-axis indicate closer relationships between pairs of Qataris. The *y*-axis shows the number of pairs with each score.
(PDF)

S2 Fig Principal components analysis of Qatari subjects alone and in combination with 1000 Genomes Phase 3 populations. Principal components (PC) analysis was conducted for **A.** n = 864 Qataris [Arab (Q1) in red, Bedouin (Q1) in pink, Persian (Q2) in blue, South Asian (Q2) in green, Sub-Saharan African (Q3) in orange]; and for **B**. Qataris in combination with 1000 Ge-nomes Phase 3 populations [5] using PLINK2 [10]. Shown is a plot of PC1 (*x*-axis) and PC2 (*y*-axis); 1000 Genomes in squares: Europeans in red, South Asians in blue, East Asians (Q2) in green, Americans in grey, Africans in orange, and Qataris in black circles.
(PDF)

S1 File Supplemental methods. (PDF)

S2 File Supplemental references. (PDF)
