# Supplementary material for: Exome sequencing-based identification of novel type 2 diabetes risk allele loci in the Qatari population
Source: PLoS One. 2018 Sep 13;13(9):e0199837. doi: 10.1371/journal.pone.0199837 (PMC6136697; doi:10.1371/journal.pone.0199837)
Supplement: S1 Table — (PDF) [file pone.0199837.s001.pdf]

Supplemental Table 1. Demographics of Type 2 Diabetes Cases and Controls

Parameters	Controls	T2D[1] cases	p value
n	290	574	
Gender (M/F)[2]	121/169	230/344	6.4×10^{-1}
Age at examination (yr) [3,4]	46 ± 9	56 ± 10	$<10^{-10}$
BMI (kg/m^2)[3,4]	31 ± 7	33 ± 7	5.5×10^{-5}
HbA1C (%)[3,4]	5.7 ± 0.4	8.4 ± 1.9	$<10^{-10}$
Glucose (mmol/L) [3,4]	5.1 ± 0.8	9.7 ± 5.0	$<10^{-10}$

[1] T2D: Type 2 diabetes
[2] Chi-square p value
[3] Data presented as mean ± standard deviation
[4] Student's t-test two-tailed distribution with unequal variance p value
