# Supplementary material for: Exome sequencing-based identification of novel type 2 diabetes risk allele loci in the Qatari population
Source: PLoS One. 2018 Sep 13;13(9):e0199837. doi: 10.1371/journal.pone.0199837 (PMC6136697; doi:10.1371/journal.pone.0199837)
Supplement: S2 Table — (PDF) [file pone.0199837.s002.pdf]

Supplemental Table 2. Results of Type 2 Diabetes Association Analyses Conducted on 864 Qataris[1]

Filter				All Protein Coding Genes[5]		T2D Genes[6]	
Function[2]	Frequency[3]	Population structure[4]	Method	Tests	Bonferroni	Tests	Bonferroni
Potentially deleterious protein coding	Low	Kinship	SKAT	9,378	6	332	0
Potentially deleterious protein coding	Low	Kinship	SVA	20,492	0	702	0

1 To identify genes and variants linked to type 2 diabetes (T2D), 2 distinct associations analyses were conducted (a gene-based test (sequence kernel association test, SKAT) and single nucleotide polymorphism (SNP)-based single variant analysis (SVA)), with 2 distinct multiple testing corrections applied. From left-to-right the columns indicate the filters applied for each analysis (frequency, function, relatives), the analysis method used (SKAT or SVA), and summaries of the analysis results with respect to all genes and known T2D genes [15]. See Supplemental Methods for full details.

2 Variants were functionally annotated using SnpEff [3], which assigned each variant to a gene and determined the potential impact of the variant on gene function based on its location, classifying variants in protein coding genes into 4 impact categories (modifier, low, moderate and high). Untranslated and intronic variants were classified as modifier impact, synonymous variants were classified as low impact, missense variants were classified as moderate impact, and nonsense or loss-of-function (LoF) variants were classified as high impact in SnpEff. Analysis was limited to potentially deleterious protein coding variants. In addition, annotation dependent depletion (CADD) scores of variant deleteriousness were calculated using CADD v.1.3 [4].

3 The annotated potentially deleterious variants in the protein coding sequence were annotated with respect to allele frequency. For each variant, the allele frequency and genotype counts in the full cohort, cases and controls was calculated using PLINK v.1.9. [10]. In addition, allele frequencies in 1000 Genomes Phase 3 v.5 [5] and ExAC v.3.1 [6] were extracted from public VCF files using VCFTools [10]. Low frequency variants are defined as those with a minor allele frequency from 0.01 to 0.1.

4 Population structure was accounted for using a kinship matrix in SKAT and SVA, calculated using EMMAX [14].

5 Shown is a summary of the SKAT or SVA analysis for all genes with at least 1 potentially deleterious variant, including the total number of tests and the total number of significant tests by Bonferroni multiple testing correction (with significant tests having $p<0.05$ divided by the number of tests).

6 Shown is a summary of the SKAT or SVA analysis for all known T2D genes [15] with at least 1 potentially deleterious variant, including the total number of tests and the total number of significant tests by Bonferroni multiple testing correction (with significant tests having $p<0.05$ divided by the number of tests).
