# Supplementary material for: Exome sequencing-based identification of novel type 2 diabetes risk allele loci in the Qatari population
Source: PLoS One. 2018 Sep 13;13(9):e0199837. doi: 10.1371/journal.pone.0199837 (PMC6136697; doi:10.1371/journal.pone.0199837)
Supplement: S3 Table — (PDF) [file pone.0199837.s003.pdf]

Supplemental Table 3. Replication of Sequence Kernel Association Test (SKAT) Significant Genes by Burden Test In An Independent Cohort[1]

Gene	SKAT test in 864 Qataris[1]		SKAT test in 12,699 individuals from T2D-GENES cohort[2]	
	Potentially deleterious variants (n)	p value	Variants (n)	p value[3]
KIF12	1	2.37×10^{-9}	0	NA
DVL1	1	3.30×10^{-7}	0	NA
EPB41L3	3	9.91×10^{-7}	4	0.81
DTNB	2	1.20×10^{-6}	0	NA
DLL1	1	3.34×10^{-6}	0	NA
CTNNB1	1	3.35×10^{-6}	0	NA

[1] SKAT test using low frequency potentially deleterious variants in all 864 Qataris.

[2] SKAT test using identical code and parameters on 12,699 exomes (6,296 cases and 6,403 controls) from the T2D-GENES cohort [20].

[3] After filtering, only *EPB41L3* contained potentially deleterious low frequency variants, hence a p value could not be calculated for the other 5 genes (marked "NA").
