# Supplementary material for: Exome sequencing-based identification of novel type 2 diabetes risk allele loci in the Qatari population
Source: PLoS One. 2018 Sep 13;13(9):e0199837. doi: 10.1371/journal.pone.0199837 (PMC6136697; doi:10.1371/journal.pone.0199837)
Supplement: S4 Table — (PDF) [file pone.0199837.s004.pdf]

Supplemental Table 4. Potentially Deleterious Low Frequency SNPs in Qataris[1,2]

										Genotype frequencies										
										Cases			Controls			Minor allele frequency[3]				
Gene	Chr	Pos	rsID	SVA p value	CADD[4]	Tran-script change	Protein change	Func-tion	Minor/ Major alleles	Hom[5] Minor (cases)	Het[6] (cases)	Hom Major (cases)	Hom Minor (con-trols)	Het (controls)	Hom Major (con-trols)	All Qataris	Cases	Controls	ExAC	1000 Genomes

Please note Supplemental Table 4 contains >20,000 rows and has been submitted as a separate EXCEL spreadsheet.

1 Single variant analysis (SVA) was conducted to identify associations between low frequency potentially deleterious variants and type 2 diabetes (T2D) using EMMAX v.10Mar2010 on all 864 Qataris, using age, gender, BMI and a kinship matrix calculated using EMMAX-KIN as covariates, identifying 6 significant genes after Bonferroni multiple testing correction (alpha = 0.05). To determine if single variants in these 6 genes were driving the SKAT association signal, the SVA p values are presented for potentially deleterious low frequency variants in all genes (n=20,642). Variants were functionally annotated using SnpEff v.4.2 using ENSEMBL v.75 gene models, and potentially deleterious variants were either missense or loss-of-function variants.

2 Shown (from left-to-right) is the gene symbol, chromosome (Chr) and position (Pos) of the variant, DbSNP v.147 rsID for the variant (or "." if novel), the SVA p value, combined annotation dependent depletion (CADD) score, transcript change (in reference-alternate allele order), protein change (in reference-alternate allele order), variant function, minor and major alleles, genotype counts for cases and controls, minor allele frequency (MAF) in all Qataris, cases, controls, ExAC, and 1000G.

3 The Qataris MAF was quantified for variants in ExAC v.0.3.1 [6] and in 1000 Genomes Phase 3 [5].

4 CADD scores were calculated for each variant to further assess the potential for deleteriousness [4].

5 Hom: Homozygous

6 Het: Heterozygous
