# Supplementary material for: Exome sequencing-based identification of novel type 2 diabetes risk allele loci in the Qatari population
Source: PLoS One. 2018 Sep 13;13(9):e0199837. doi: 10.1371/journal.pone.0199837 (PMC6136697; doi:10.1371/journal.pone.0199837)
Supplement: S5 Table — (PDF) [file pone.0199837.s005.pdf]

Supplemental Table 5. Known Protein Coding Loci Linked to Type 2 Diabetes[1,2]

Gene	Chr	Pos	rsID	SVA p value	CADD[4]	Transcript change	Protein change	Function	Minor/ Major Alleles[5]	Genotype frequencies: Cases Hom Minor (cases)[6]	Cases Het (cases)[7]	Cases Hom Major (cases)	Controls Hom Minor (controls)	Controls Het (controls)	Controls Hom Major (controls)	Minor allele frequency[3]: Qataris	Cases	Controls	ExAC	1000 Genomes
MACF1	1	39835817	rs2296172	0.078	9.0	c.13165A>G	p.Met4389Val	Missense	**G**/A	10	135	429	6	54	230	0.1279	0.1350	0.1138	0.1830	0.1182
PPARG	3	12393125	rs1801282	0.445	20.6	c.34C>G	p.Pro12Ala	Missense	G/**C**	0	63	511	0	27	263	0.0521	0.0549	0.0466	0.1100	0.0703
WFS1	4	6303022	rs1801214	0.962	0.9	c.1500C>T	p.Asn500Asn	Synonymous	C/**T**	90	266	218	43	140	107	0.3889	0.3885	0.3897	0.3410	0.2706
KCNK16	6	39284050	rs1535500	0.099	1.8	c.830C>A	p.Ala277Glu	Missense	G/**T**	57	244	273	41	127	122	0.3281	0.3118	0.3603	0.5160	0.4111
ANK1	8	41519462	rs515071	0.036	3.2	c.5479-3T>C		Intron	A/**G**	31	186	357	26	106	158	0.2350	0.2160	0.2724	0.7790	0.7863
KCNJ11	11	17408630	rs5215	0.080	12.4	c.1009G>A	p.Val337Ile	missense	**C**/T	35	191	348	17	89	184	0.2222	0.2274	0.2121	0.6450	0.7306

1 Single variant analysis (SVA) was conducted to identify associations between low frequency potentially deleterious variants and type 2 diabetes (T2D) using EMMAX v.10Mar2010 on all 864 Qataris, using age, gender, BMI and a kinship matrix calculated using EMMAX-KIN as covariates. To determine if known T2D loci replicate in Qatar, variants present in prior reports of T2D variants [15] were extracted from the set of n=295,515. Shown are the n=6 of n=81 known T2D variants confidently genotyped in this study. Variants were functionally annotated using SnpEff v.4.2 using ENSEMBL v.75 gene models, and potentially deleterious variants were either missense or loss-of-function variants.

2 Shown (from left-to-right) is the gene symbol, chromosome (Chr) and variant position (Pos), DbSNP v.147 rsID for the variant (or "." if novel), the SVA p value, combined annotation dependent depletion (CADD) score, transcript change (in reference-alternate allele order), protein change (in reference-alternate allele order), variant function, minor and major alleles, genotype counts for cases and controls, minor allele frequency in all Qataris, cases, controls, ExAC, and 1000G. The table includes all n=20,642 low frequency potentially deleterious variants.

3 The Qatari minor allele frequency was quantified for variants in ExAC v.0.3.1 [6] and in 1000 Genomes Phase 3 [5].

4 CADD scores were calculated for each variant to further assess the potential for deleteriousness [4].

5 Risk alleles indicated in bold, based on prior reports [21–25].

6 Hom: Homozygous

7 Het: Heterozygous
