# Supplementary material for: Exome sequencing-based identification of novel type 2 diabetes risk allele loci in the Qatari population
Source: PLoS One. 2018 Sep 13;13(9):e0199837. doi: 10.1371/journal.pone.0199837 (PMC6136697; doi:10.1371/journal.pone.0199837)
Supplement: S6 Table — (PDF) [file pone.0199837.s006.pdf]

Supplemental Table 6. Power Calculation for Known Protein Coding Loci Linked to Type 2 Diabetes[1]

Gene	*MACF1*	*PPARG*	*WFS1*	*KCNK16*	*ANK1*	*KCNJ11*
Variant	rs2296172	rs1801282	rs1801214	rs1535500	rs515071	rs5215
High risk allele frequency	0.13	0.95	0.61	0.67	0.77	0.22
Disease prevalence	0.22	0.22	0.22	0.22	0.22	0.22
Genotype relative risk Aa	1.28	1.16	1.13	1.01	1.18	1.14
Genotype relative risk AA	1.64	1.35	1.28	1.02	1.39	1.30
D-prime	1.00	1.00	1.00	1.00	1.00	1.00
Marker allele frequency (B)	0.13	0.95	0.61	0.67	0.77	0.22
Number of cases	574	574	574	574	574	574
Control:case ratio	0.51	0.51	0.51	0.51	0.51	0.51
User defined type I error rate	0.05	0.05	0.05	0.05	0.05	0.05
User defined power	0.80	0.80	0.80	0.80	0.80	0.80
Samples for 80% power at alpha 0.05	949	6,882	1,965	327,729	1,542	2,324
Power	0.59	0.13	0.33	0.05	0.40	0.29

[1] Power calculation for known loci was conducted using Purcell's Genetic Power Calculator [21–26].
