# Supplementary figures and images for: Exome sequencing-based identification of novel type 2 diabetes risk allele loci in the Qatari population
Source: PLoS One. 2018 Sep 13;13(9):e0199837. doi: 10.1371/journal.pone.0199837 (PMC6136697; doi:10.1371/journal.pone.0199837)

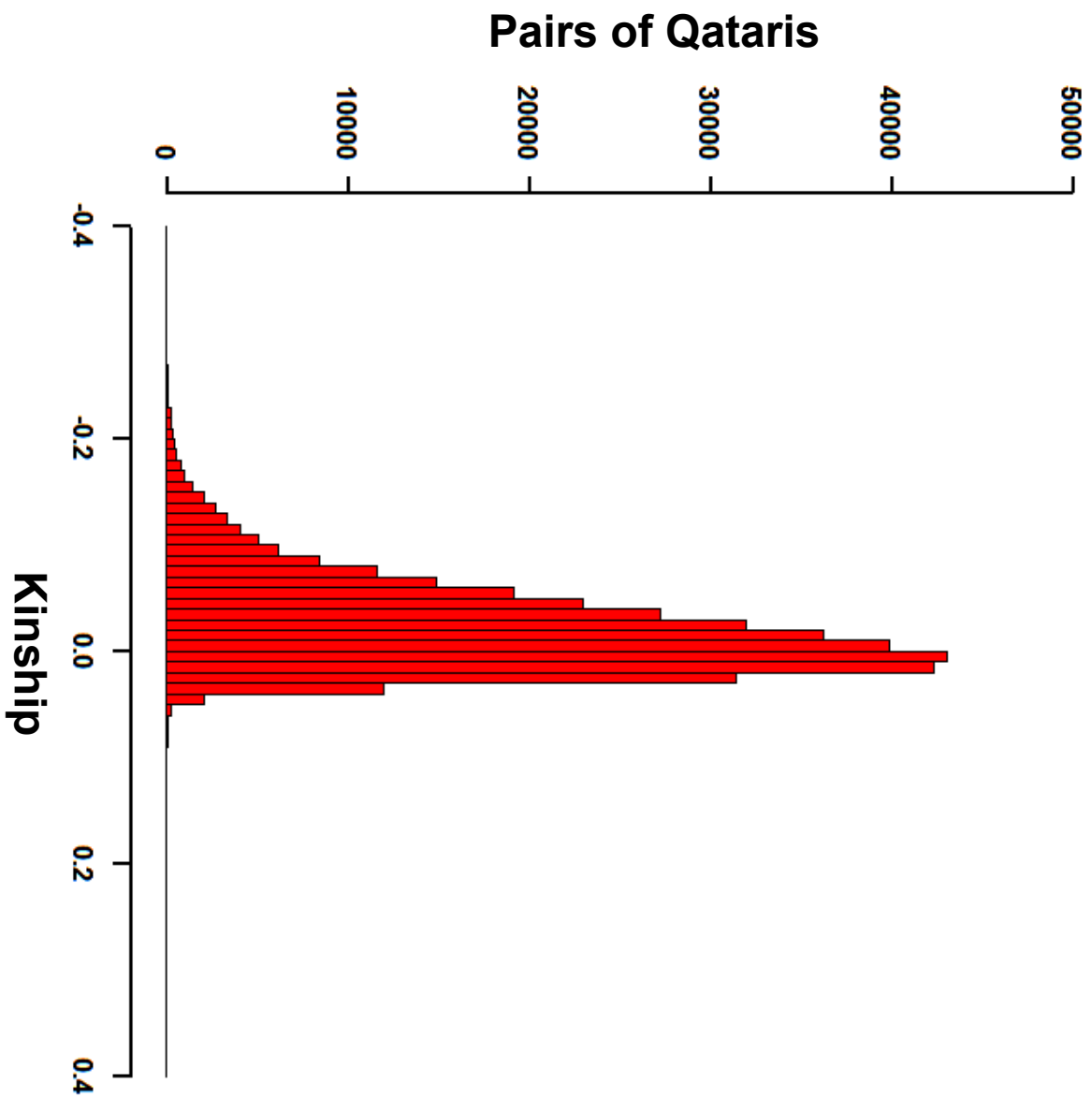

Supplement: S1 Fig — In order to account for population structure and relatedness in the analysis, kinship was calculated for each pair of Qataris (n = 864 individuals) using KING v.2 [27]. Shown is the frequency distribution of kinship scores, where higher numbers on the x-axis indicate closer relationships between pairs of Qataris. The y-axis shows the number of pairs with each score. (PDF) [file pone.0199837.s007.pdf]
