# Supplementary material for: Exome sequencing-based identification of novel type 2 diabetes risk allele loci in the Qatari population
Source: PLoS One. 2018 Sep 13;13(9):e0199837. doi: 10.1371/journal.pone.0199837 (PMC6136697; doi:10.1371/journal.pone.0199837)
Supplement: S2 Fig — Principal components (PC) analysis was conducted for A. n = 864 Qataris [Arab (Q1) in red, Bedouin (Q1) in pink, Persian (Q2) in blue, South Asian (Q2) in green, Sub-Saharan African (Q3) in orange]; and for B. Qataris in combination with 1000 Ge-nomes Phase 3 populations [5] using PLINK2 [10]. Shown is a plot of PC1 (x-axis) and PC2 (y-axis); 1000 Genomes in squares: Europeans in red, South Asians in blue, East Asians (Q2) in green, Americans in grey, Africans in orange, and Qataris in black circles. (PDF) [file pone.0199837.s008.pdf]

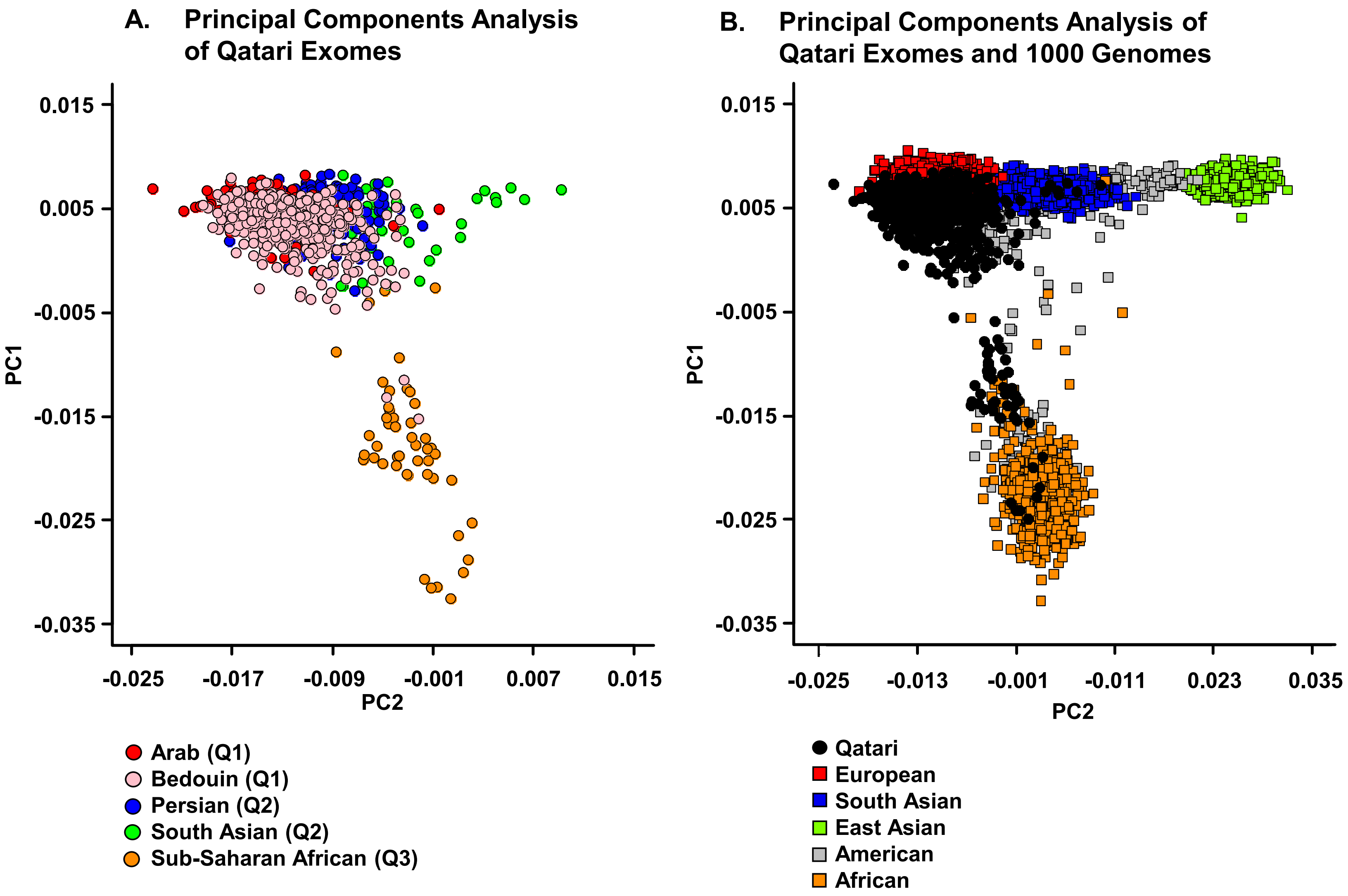
A. Principal Components Analysis of Qatari Exomes
0.015
0.005
-0.005
-0.015
-0.025
-0.035
PC1
-0.025
-0.017
-0.009
-0.001
0.007
0.015
PC2
Arab (Q1)
Bedouin (Q1)
Persian (Q2)
South Asian (Q2)
Sub-Saharan African (Q3)
B. Principal Components Analysis of Qatari Exomes and 1000 Genomes
0.015
0.005
-0.005
-0.015
-0.025
-0.035
PC1
-0.025
-0.013
-0.001
-0.011
0.023
0.035
PC2
Qatari
European
South Asian
East Asian
American
African

S2 Fig
