# Supplementary material for: Exome sequencing-based identification of novel type 2 diabetes risk allele loci in the Qatari population
Source: PLoS One. 2018 Sep 13;13(9):e0199837. doi: 10.1371/journal.pone.0199837 (PMC6136697; doi:10.1371/journal.pone.0199837)
Supplement: S1 File — (PDF) [file pone.0199837.s009.pdf]

Supplemental Methods

Study Population

Under protocols approved by the Institutional Review Boards of Hamad Medical Corporation (HMC) and Weill Cornell Medical College Qatar (WCMC-Q), subjects were recruited from HMC clinics and written informed consent obtained. A total of 864 subjects (574 cases with type 2 diabetes (T2D) and 290 controls) were included in the study (S1 Table). T2D was diagnosed based on the American Diabetes Association (ADA) criteria, including a fasting plasma glucose ≥126 mg/dL and/or 2 hr plasma glucose ≥200 mg/dL during an oral glucose tolerance test, and/or HbA1C ≥ 6.5% [1].

A questionnaire, physical examination and medical record review were performed and blood collected from each subject. Demographic data including age and gender, personal and family history of diabetes was recorded along with self-reported history of gestational diabetes, duration of diabetes and medication use including duration of insulin therapy if applicable. Physical characteristics such as height and weight were collected and body mass index (BMI) calculated. Laboratory tests included HbA1C and glucose levels.

All subjects were over the age of 30 with a family history of a minimum three generations of ancestry in Qatar. Cases were excluded if any of the following were present: history of type 1 diabetes, maturity onset diabetes of the young (MODY), maternally inherited diabetes and deafness syndrome (MIDD), a first degree relative with type 1 diabetes, or secondary diabetes.

Sequencing and Variant Detection

Sequencing was performed at the New York Genome Center (NYGC). Exome DNA enrichment was performed using Agilent SureSelect Human All Exon Kit version (V) 5 (Agilent Technologies, Santa Clara, CA) hybrid capture targets for 51 MB of coding regions, with sequencing on the Illumina HiSeq 2500 platform (Illumina Inc., San Diego, CA) using a full lane

for each exome. A call set for all 864 exomes was produced by simultaneous genotyping using the GATK UnifiedGenotyper algorithm [2]. Data quality was optimized and verified based on multiple metrics described in detail below. Variants were categorized based on minor allele frequency (MAF), into singleton (MAF <0.001, n=46,874), rare (MAF 0.001 to 0.01, n=190,771, including n=64,258 doubletons), low-frequency (MAF 0.01 to 0.1, n=58,671), and common (MAF >0.1, n=46,073). Singleton variants were excluded due to their expected enrichment for sequencing artifacts, leaving n=295,515 variants for analysis.

Variants in protein coding genes were identified using SnpEff v.4.2 [3], which uses ENSEMBL v.75 gene models to assign variants to genes, determine their functional region and impact on the gene. SnpEff classified variants in protein coding genes into 4 impact categories (modifier, low, moderate and high). Untranslated and intronic variants were classified as modifier impact, synonymous variants were classified as low impact, missense variants were classified as moderate impact, and nonsense or loss-of-function (LoF) variants were classified as high impact. Three successive functional filters were applied to the data. The first functional filter kept variants in the "protein coding transcript", which included all 4 impact categories, or from a molecular perspective all variants in the open reading frame or unprocessed mRNA transcript (n=295,515). The second functional filter kept variants in the "protein coding sequence", which included low, moderate and high impact variants, or from a molecular perspective all variants in the transcribed (amino-acid coding) sequence (n=206,186). The third functional filter kept "potentially deleterious protein coding" variants, or from a molecular perspective all variants with a moderate to high potential for altering protein structure and function (n=111,358). In addition, quantitative scores of deleteriousness were calculated using CADD v.1.3 [4]. The variants were annotated with respect to allele frequency in the cohort, 1000 Genomes Phase 3 v.5 [5] and ExAC v.3.1 [6], calculated using VCFTools v.0.1.14 [7].

Data Quality Control

The mapped read data in BAM format and genotype data in population VCF format was extensively analyzed and curated for quality. BAM files of paired-end exome sequence reads mapped to the GRCh37 human reference genome were prepared for variant detection using GATK Best Practices [8], including removal of duplicate reads, realignment across known indels, and recalibration of base quality scores. Mean coverage depth was 60*. Gender was verified for each BAM by calculating the ratio of coverage depth in ChrX:ChrY, with all males having a ratio near 1:1 and all females having a depth ratio significantly higher. Variant genotype calling was limited to sites in the Agilent SureSelect V5 targets, and variants observed with a Phred-scaled variant quality score of 30 (Q30) or higher. All sites kept had an average of 0.1% missing genotypes across samples, and individual exomes had an average of 0.1% missing genotype rate. Genotypes were verified to be over 99% concordant with whole genome sequencing data for a subset of the samples in a prior publication [9]. To exclude outliers, a principal components (PC) analysis was conducted on a subset of the n=295,515 non-singleton variants pruned for linkage equilibrium using PLINK v.1.9 [10] "indep-pairwise" command (window 1000 SNPs, step size 25 SNPs, maximum r^2 0.25). PCs were calculated using PLINK v.1.9, and for the first 10 PCs all samples were confirmed to be within 1 standard deviation (SD) of the mean (S2A Fig). PCs were also calculated in combination with 1000 Genomes Phase 3 populations using ancestry-informative variants identified in a prior study [9] as further confirmation of population structure (S2B Fig).

In the PC plot with 1000 Genomes Phase 3, all Qatari individuals were visually confirmed to overlap with individuals of similar ancestries, based on prior studies of population structure that classify Qataris as having predominantly Arab, Bedouin, Persian, South Asian, or Sub-Saharan African ancestry [9,11]. While early studies classified these individuals into 3 mac-

ro-populations (Arab/Bedouin or Q1, Persian/South Asian or Q2, Sub-Saharan African or Q3), recent studies comparing n=1376 Qatari exomes (including the 864 individuals in this study) to deeper reference panels [12] have allowed for higher resolution ancestry inference of Qataris. Hence, the PC plots in this study are reproduced from the Supplemental Figures of Fakhro et al [9] (https://arxiv.org/abs/1805.03233). Based on the ancestry inference in this prior study, the 864 Qataris in the current study included n=605 Q1 (n=440 Bedouin and n=165 Arab), n=210 Q2 (n=150 Persian and n=60 South Asian) and n=49 Q3 (Sub-Saharan African).

Statistical Analysis

To identify genes linked to diabetes most effectively, the analysis was limited to low frequency (MAF 0.01 to 0.1) potentially deleterious variants (annotated as “moderate” or “high” impact by SnpEff). To exhaustively search for genes and variants linked to T2D in Qatar, a total of 4 distinct association analyses were conducted on the whole-exome sequence data genotypes, including gene-based analysis (sequence kernel association test, SKAT) [13], and variant-based (single variant analysis, SVA) [14], with Bonferroni multiple testing correction for all genes and known T2D genes [15]. The genetic models tested included an association test for each variant after applying prior filters (SVA), and an association test for each gene (SKAT) containing at least 1 variant after filtering. The SVA was conducted using EMMAX [14], taking as covariates age, gender, BMI and the kinship matrix calculated by EMMAX-KIN. The SKAT test was conducted using the SKAT v.1.2.2 (https://cran.r-project.org/web/packages/SKAT/) library in R v.3.3.2. (https://cran.r-project.org/). Two gene set filters were considered, one consisting of all protein coding genes tested, and a second consisting of the subset of tested genes that overlap with the list of 634 genes linked to T2D (Supplemental Table 20 of Fuchsberg *et al* [15]). These gene sets were used for multiple testing corrections by the Bonferroni method [16] with alpha = 0.05. Population structure was accounted for in the SKAT and SVA analysis using the kinship

matrix, which is effective for both near and distant relationships, as in this cohort. Prior studies have shown a high degree of consanguinity within the Q1 and Q2 sub-populations [17] comparable to other Middle Eastern populations such as Jordanians and Palestinians, with low consanguinity in the Q3 subpopulation, comparable to other African populations [17]. Kinship analysis was conducted using EMMAX-KIN v.10Mar2010 [14], and the distribution of kinship was plotted and confirmed to demonstrate high degrees of relatedness among Q1 and Q2, as previously reported for 1,376 Qataris, including the 864 in this study [9].

The results of the SVA and SKAT statistical analyses were evaluated with respect to significance and inflation using Manhattan plots and QQ plots. A Manhattan plot of the –log10 p values for the SKAT results was plotted, with variants ordered by chromosome and gene position, with one point in the Manhattan plot per gene. Genes passing the Bonferroni multiple testing correction were labelled with gene symbol. The QQ plots were produced in R for both SKAT and SVA, where the SKAT results included a p value per gene and the SVA results included a p value per variant. In order to assess for p value inflation due to population structure or polygenic disease risk [18], the lambda value was calculated for both the SVA and SKAT p value distributions. The lambda was calculated by converting the SVA and SKAT and p value distributions to Chi-square statistic distributions in R, and then calculating and dividing the median statistic by 0.456 [19].

Replication

To replicate the observed associations in a distinct cohort, SKAT p values were calculated for 12,699 exomes (6296 cases and 6403 controls) in the T2D-Genes cohort [20], downloaded with permission from the European Genome Archive (dataset ID EGAS00001001460, https://www.ebi.ac.uk/ega/studies/EGAS00001001460). Exome and phenotype data was downloaded, and analysis conducted on individuals where all covariates (age, gender, BMI) were

available. Analysis was conducted using the identical code and filter parameters as for the Qatari data (limited to low-frequency potentially deleterious variants), using a kinship matrix to control for relatedness and population structure. Due to the computational limitation of whole-exome analysis using SKAT, p values were calculated for only 6 the Qatari SKAT-significant genes by extracting variants within these genes (Table 1) from the full VCF.

Investigation of Known T2D Loci

The Qatari exome data was queried for n=81 variants recently reported in known T2D loci [15]. Confidently genotyped variants from this list (from n=295,515 variants present before functional and frequency filtering) were extracted. For each variant, the risk allele was identified from primary literature, and the prevalence in Qataris cases and controls was assessed.

Data Sharing

All sequence read data and individual and population VCF files were submitted to the Sequence Read Archive (SRA) section of the NCBI SRA database (SRA accession #SRP061463, BioProject 288292). In addition, phenotype and covariate data and the population VCF was made available through our website http://geneticmedicine.weill.cornell.edu/genome.html. Furthermore, an analysis pipeline written in Python was shared *via* GitHub (https://github.com/juansearch/_WAS) for replication of the results in this study.
