# Supplementary material for: Exome sequencing-based identification of novel type 2 diabetes risk allele loci in the Qatari population
Source: PLoS One. 2018 Sep 13;13(9):e0199837. doi: 10.1371/journal.pone.0199837 (PMC6136697; doi:10.1371/journal.pone.0199837)
Supplement: S2 File — (PDF) [file pone.0199837.s010.pdf]

## Supplemental References


1. Standards of medical care in diabetes--2013. Diabetes Care 36 Suppl 1: S11-S66.

2. DePristo MA, Banks E, Poplin R, Garimella KV, Maguire JR, Hartl C et al. (2011) A framework for variation discovery and genotyping using next-generation DNA sequencing data. Nat Genet 43: 491-498.

3. Cingolani P, Platts A, Wang lL, Coon M, Nguyen T, Wang L et al. (2012) A program for annotating and predicting the effects of single nucleotide polymorphisms, SnpEff: SNPs in the genome of Drosophila melanogaster strain w1118; iso-2; iso-3. Fly (Austin ) 6: 80-92. 19695 [pii];10.4161/fly.19695 [doi].

4. Kircher M, Witten DM, Jain P, O'Roak BJ, Cooper GM, Shendure J (2014) A general framework for estimating the relative pathogenicity of human genetic variants. Nat Genet 46: 310-315.

5. Auton A, Brooks LD, Durbin RM, Garrison EP, Kang HM, Korbel JO et al. (2015) A global reference for human genetic variation. Nature 526: 68-74. nature15393 [pii];10.1038/nature15393 [doi].

6. Karczewski KJ, Weisburd B, Thomas B, Solomonson M, Ruderfer DM, Kavanagh D et al. (2017) The ExAC browser: displaying reference data information from over 60 000 exomes. Nucleic Acids Res 45: D840-D845. gkw971 [pii];10.1093/nar/gkw971 [doi].

7. Danecek P, Auton A, Abecasis G, Albers CA, Banks E, DePristo MA et al. (2011) The variant call format and VCFtools. Bioinformatics 27: 2156-2158. btr330 [pii];10.1093/bioinformatics/btr330 [doi].

8. McKenna A, Hanna M, Banks E, Sivachenko A, Cibulskis K, Kernytsky A et al. (2010) The Genome Analysis Toolkit: a MapReduce framework for analyzing next-generation DNA sequencing data. Genome Res 20: 1297-1303.

9. Fakhro KA, Staudt MR, Ramstetter MD, Robay A, Malek JA, Badii R et al. (2016) The Qatar genome: a population-specific tool for precision medicine in the Middle East. Hum Genome Var 3: 16016. 10.1038/hgv.2016.16 [doi].

10. Chang CC, Chow CC, Tellier LC, Vattikuti S, Purcell SM, Lee JJ (2015) Second-generation PLINK: rising to the challenge of larger and richer datasets. Gigascience 4: 7.

11. Hunter-Zinck H, Musharoff S, Salit J, Al-Ali KA, Chouchane L, Gohar A et al. (2010) Population genetic structure of the people of Qatar. Am J Hum Genet 87: 17-25. S0002-9297(10)00266-1 [pii];10.1016/j.ajhg.2010.05.018 [doi].

12. Lazaridis I, Patterson N, Mittnik A, Renaud G, Mallick S, Kirsanow K et al. (2014) Ancient human genomes suggest three ancestral populations for present-day Europeans. Nature 513: 409-413.

13. Lee S, Emond MJ, Bamshad MJ, Barnes KC, Rieder MJ, Nickerson DA et al. (2012) Optimal unified approach for rare-variant association testing with application to small-sample case-control whole-exome sequencing studies. Am J Hum Genet 91: 224-237.

14. Kang HM, Sul JH, Service SK, Zaitlen NA, Kong SY, Freimer NB et al. (2010) Variance component model to account for sample structure in genome-wide association studies. Nat Genet 42: 348-354. ng.548 [pii];10.1038/ng.548 [doi].

15. Fuchsberger C, Flannick J, Teslovich TM, Mahajan A, Agarwala V, Gaulton KJ et al. (2016) The genetic architecture of type 2 diabetes. Nature . nature18642 [pii];10.1038/nature18642 [doi].

16. Bland JM, Altman DG (1995) Multiple significance tests: the Bonferroni method. BMJ 310: 170.

17. Rodriguez-Flores JL, Fakhro K, Agosto-Perez F, Ramstetter MD, Arbiza L, Vincent TL et al. (2016) Indigenous Arabs are descendants of the earliest split from ancient Eurasian populations. Genome Res 26: 151-162. gr.191478.115 [pii];10.1101/gr.191478.115 [doi].

18. Yang J, Weedon MN, Purcell S, Lettre G, Estrada K, Willer CJ et al. (2011) Genomic inflation factors under polygenic inheritance. Eur J Hum Genet 19: 807-812. ejhg201139 [pii];10.1038/ejhg.2011.39 [doi].

19. Hao K, Li C, Rosenow C, Wong WH (2004) Detect and adjust for population stratification in population-based association study using genomic control markers: an application of Affymetrix Genechip Human Mapping 10K array. Eur J Hum Genet 12: 1001-1006. 10.1038/sj.ejhg.5201273 [doi];5201273 [pii].

20. Flannick J, Fuchsberger C, Mahajan A, Teslovich TM, Agarwala V, Gaulton KJ et al. (2017) Sequence data and association statistics from 12,940 type 2 diabetes cases and controls. Sci Data 4: 170179. sdata2017179 [pii];10.1038/sdata.2017.179 [doi].

21. Altshuler D, Hirschhorn JN, Klannemark M, Lindgren CM, Vohl MC, Nemesh J et al. (2000) The common PPARgamma Pro12Ala polymorphism is associated with decreased risk of type 2 diabetes. Nat Genet 26: 76-80. 10.1038/79216 [doi].

22. Dayeh TA, Olsson AH, Volkov P, Almgren P, Ronn T, Ling C (2013) Identification of CpG-SNPs associated with type 2 diabetes and differential DNA methylation in human pancreatic islets. Diabetologia 56: 1036-1046.

23. Imamura M, Maeda S, Yamauchi T, Hara K, Yasuda K, Morizono T et al. (2012) A single-nucleotide polymorphism in ANK1 is associated with susceptibility to type 2 diabetes in Japanese populations. Hum Mol Genet 21: 3042-3049. dds113 [pii];10.1093/hmg/dds113 [doi].

24. Sakai K, Imamura M, Tanaka Y, Iwata M, Hirose H, Kaku K et al. (2013) Replication study for the association of 9 East Asian GWAS-derived loci with susceptibility to type 2 diabetes in a Japanese population. PLOS ONE 8: e76317. 10.1371/journal.pone.0076317 [doi];PONE-D-13-17352 [pii].

25. Zeggini E, Weedon MN, Lindgren CM, Frayling TM, Elliott KS, Lango H et al. (2007) Replication of genome-wide association signals in UK samples reveals risk loci for type 2 diabetes. Science 316: 1336-1341. 1142364 [pii];10.1126/science.1142364 [doi].

26. Purcell S, Cherny SS, Sham PC (2003) Genetic Power Calculator: design of linkage and association genetic mapping studies of complex traits. Bioinformatics 19: 149-150.

27. Manichaikul A, Mychaleckyj JC, Rich SS, Daly K, Sale M, Chen WM (2010) Robust relationship inference in genome-wide association studies. Bioinformatics 26: 2867-2873.
